# PD49 Developing Evidence-Based Optimal Testing Strategies To Monitor Long-Term Conditions In Primary Care

**DOI:** 10.1017/S0266462324003088

**Published:** 2025-01-07

**Authors:** Martha Elwenspoek, Rachel O’Donnell, Katie Charlwood, Alice Malpass, Mary Ward, Howard Thom, Jonathan Banks, Clare Thomas, Hayley Jones, Jonathan Sterne, Francesco Palma, Christina Stokes, Alastair Hay, Jessica Watson, Penny Whiting

## Abstract

**Introduction:**

Most patients with long-term conditions (LTC) receive regular blood tests to monitor disease progression and response to treatment and to detect complications. There is currently no robust evidence to inform recommendations on monitoring. Creating this evidence base is challenging because the benefits and harms of testing are dependent on what is done in response to the test results.

**Methods:**

We identified a list of commonly used tests. We defined a series of filtering questions to determine whether there was evidence to support the rationale of monitoring, such as “Can the general practitioner do anything in response to an abnormal test result?” Through a series of rapid reviews we identified evidence to answer each question. The evidence was presented at a consensus meeting where clinicians and patients voted for inclusion, exclusion, or further analysis. A process evaluation was performed alongside this. Further analyses were performed using routinely collected healthcare data and by performing incidence analyses, emulating randomized controlled trials (RCTs), and modeling disease progression.

**Results:**

We tested this methodology on three common LTCs: chronic kidney disease (CKD), type 2 diabetes mellitus (T2DM), and hypertension. We found sufficient evidence to include hemoglobin A1C and estimated glomerular filtration rate (eGFR) for monitoring patients with T2DM; hemoglobin and eGFR for patients with CKD; and eGFR for patients with hypertension. The consensus panel excluded four tests, while 10 tests were selected for further analysis. The emulated RCTs will investigate the effect of regular monitoring with certain tests on health outcomes among routinely monitored patients. In addition, we will investigate the signal-to-noise ratio of each test over time using a modeling approach.

**Conclusions:**

The cost effectiveness of the evidence-based testing panels needs to be tested in clinical practice. We are currently developing an intervention package and are planning to run a feasibility trial. This program of work has the potential to change how LTCs are monitored in primary care, ultimately improving patient outcomes and leading to more efficient use of healthcare resources.

